# Explainable feature selection and deep learning based emotion recognition in virtual reality using eye tracker and physiological data

**DOI:** 10.3389/fmed.2024.1438720

**Published:** 2024-09-12

**Authors:** Hadeel Alharbi

**Affiliations:** College of Computer Science and Engineering, University of Hail, Ha'il, Saudi Arabia

**Keywords:** emotion recognition, deep learning, machine learning, virtual reality, EEG, explainable artificial intelligence (XAI)

## Abstract

Emotional recognition is a way of detecting, evaluating, interpreting, and responding to others' emotional states and feelings, which might range from delight to fear to disgrace. There is increasing interest in the domains of psychological computing and human-computer interface (HCI), especially Emotion Recognition (ER) in Virtual Reality (VR). Human emotions and mental states are effectively captured using Electroencephalography (EEG), and there has been a growing need for analysis in VR situations. In this study, we investigated emotion recognition in a VR environment using explainable machine learning and deep learning techniques. Specifically, we employed Support Vector Classifiers (SVC), K-Nearest Neighbors (KNN), Logistic Regression (LR), Deep Neural Networks (DNN), DNN with flattened layer, Bi-directional Long-short Term Memory (Bi-LSTM), and Attention LSTM. This research utilized an effective multimodal dataset named VREED (VR Eyes: Emotions Dataset) for emotion recognition. The dataset was first reduced to binary and multi-class categories. We then processed the dataset to handle missing values and applied normalization techniques to enhance data consistency. Subsequently, explainable Machine Learning (ML) and Deep Learning (DL) classifiers were employed to predict emotions in VR. Experimental analysis and results indicate that the Attention LSTM model excelled in binary classification, while both DNN and Attention LSTM achieved outstanding performance in multi-class classification, with up to 99.99% accuracy. These findings underscore the efficacy of integrating VR with advanced, explainable ML and DL methods for emotion recognition.

## 1 Introduction

Emotion plays a crucial role in interpersonal interactions, knowledge insight, perception, and daily life activities (1, 2). Understanding emotions is essential for effective social interaction, making Affective Computing (AC) methods vital for enhancing human-computer interaction by identifying and assessing human emotional states (3). The intersection of technology, emotion, and human experience has led to significant breakthroughs, particularly in VR and emotion recognition, which continue to advance rapidly.

ER involves detecting human emotions using features extracted from various datasets, followed by ML and DL methods. Traditional methods use text, speech, body posture, facial expressions, and physiological signals like Electrocardiograms (ECGs) and Electroencephalograms (EEGs) (4). EEGs are particularly advantageous in ER research due to their cost-effectiveness, objectivity, temporal precision, and non-invasive nature (5). While classical ER studies often use 2D stimuli, VR has emerged as a superior medium for eliciting genuine emotional responses, offering immersive experiences that enhance the authenticity of emotion experiments (6).

VR's application extends to fields such as education (7), architectural design (8), and virtual tourism, especially amid the pandemic (9). In the field of education, VR facilitates immersive learning experiences that can lead to better understanding and retention of complex subjects (10). In medicine, VR is used for patient rehabilitation and surgical training, providing a safe and controlled environment for practice and recovery (11).

Recent advancements in Natural Language Processing (NLP) and computer vision have furthered the capabilities of ER systems. NLP techniques allow for the analysis of textual data to infer emotional states, while computer vision methods analyze facial expressions and body movements (12). The fusion of these modalities with physiological signals such as EEG and ECG enhances the accuracy and robustness of ER systems (13). The continuous evolution of VR and ER technologies promises to deliver more sophisticated and intuitive interactions between humans and machines, facilitating a range of applications from mental health assessments to customer service automation (14). The integration of these technologies into everyday devices and applications signifies a future where technology can seamlessly understand and respond to human emotions, improving both user experience and functionality.

### 1.1 Research motivation and contributions

Despite significant advancements, emotion recognition in immersive technologies like VR remains challenging. There is a growing need to develop robust methods that can accurately detect and respond to users' emotional states in real-time, enhancing user experiences across various applications. Leveraging ML and DL approaches to detect user emotions can significantly enhance user experiences by adjusting interactions and visuals based on emotional feedback. This study proposes a binary and multi-class approach using ML classifiers (KNN, SVC, LR) and DL classifiers (DNN, DNN with a flattened layer, Bi-LSTM, and Attention LSTM) for emotion recognition in VR, utilizing eye-tracking and physiological data. Compared to previous techniques, our approach demonstrates improved performance and offers a scalable solution for real-time emotion recognition in VR environments. The main findings of this study are outlined below.

- Propose an explainable feature selection and deep learning-based approach for binary and multi-class detection and categorization of emotions in virtual reality using eye tracker and physiological data by utilizing a range of ML classifiers, including K-Nearest Neighbors (KNN), Support Vector Classifier (SVC), and Logistic Regression (LR), as well as deep learning (DL) classifiers such as Deep Neural Networks (DNN), DNN with flattened layers, Bi-directional Long Short-Term Memory (Bi-LSTM), and Attention LSTM. These models are trained and evaluated using eye tracker and physiological data, aiming to improve emotional recognition accuracy in immersive VR environments.
- Comprehensive data preprocessing techniques are employed, including handling missing values, data splitting, and z-score normalization. These steps are crucial to ensure data quality and model performance consistency across different classifiers. Evaluation metrics were rigorously applied to compare and analyze the performance of both ML and DL models.
- The Attention LSTM model demonstrated exceptional performance in both binary and multi-class classification tasks, achieving up to 99.99% accuracy. This highlights its capability to capture temporal dependencies and contextual information in VR data effectively. Comparative analyses with existing techniques showed superior performance of the proposed approach, underscoring its potential for advancing emotion recognition methodologies in virtual reality.

### 1.2 Research organization

The next sections of this article are carefully structured to help readers in comprehending the study's methodology. Background information and an overview of the results of the current investigation are presented in Section 2. Section 3 goes into great depth on the technical aspects of the suggested framework, the dataset preliminaries, data preprocessing techniques, and ML and DL models. In Section 4, the experimental data are provided together with a summary of the majority of the study's conclusions. Section 6 of the research offers a comprehensive analysis and findings derived from the suggested work. This part also presents the future directions of the current work.

## 2 Related work

In this section, we examine earlier research that applied machine learning and deep learning classifiers for emotion recognition. The study Rahman et al. (15) explored machine learning techniques, using publicly available electroencephalogram and heart rate variability datasets to predict arousal levels. Detecting anxiety-induced arousal enables timely interventions to manage distress. They discuss methods for effective machine learning model selection and propose a tailored pipeline for VRET that is applicable to various contexts requiring arousal detection. Talaat (16) introduces an emotion identification framework tailored for autistic children. An Enhanced Deep Learning (EDL) technique leveraging convolutional neural networks is proposed for emotion classification, with the framework benefiting from fog and IoT technologies to minimize latency and enhance real-time detection and location awareness. Results demonstrate EDL's superior performance, achieving 99.99% accuracy, with genetic algorithms utilized to optimize hyperparameters for the CNN.

Yang and Ismail (17) presented a Multimodal Fusion Deep LSTM (MFD-LSTM) model to introduce a fresh concept for tea packaging. By using this method, tea packaging becomes more dynamic and immersive, appealing to all senses and highlighting the combination of VR, emotion recognition, and the MFD-LSTM model. Tea packaging may now dynamically tell brand tales and elicit strong feelings from customers thanks to this connection. With its ability to integrate many sensory inputs at once, the MFD-LSTM model influences the unfolding VR experience by facilitating real-time emotion identification. The goal of this study is to create a multimodal and emotionally engaging relationship between tea brands and customers by promoting the widespread use of interactive tea packaging that makes use of virtual reality, emotion recognition, and the MFD-LSTM model. The analysis results show that the suggested MFD-LSTM model is useful for assessing emotions and improving packaging performance. Emotion prediction utilizing Heart Rate (HR) signals through common classifiers like SVM, KNN, and RF in a VR setting is explored in Bulagang et al. (18). Experimentation with Empatica E4 wristbands and VR headsets showed promising results, with SVM, KNN, and RF achieving 100% accuracy in intra-subject classification and moderate accuracy in inter-subject classification. This highlights the potential of using HR classification for emotion prediction in various VR applications, including interactive gaming, affective entertainment, and VR health rehabilitation.

An emotive cue detection method for an adaptive music system (LitSens) in virtual reality environments is presented by Ibá nez et al. (19), with the goal of improving immersion. A hybrid one-dimensional convolutional neural network and a multi-layer perceptron-based system are the two neural network-based iterations that are examined. For soundtrack adaption, the second iteration is preferred as it is more accurate but takes longer to recognize fear than the first. Positive results are generally obtained from an experiment that supports the gesture recognizer's ability to identify fear in participants. Savchenko et al. (20) article delve into the analysis of students' behavior within the e-learning environment, proposing a novel approach centered around video facial processing. Initially, techniques such as face detection, tracking, and clustering are employed to extract sequences of individual students' faces. This network undergoes pre-training for face identification and is finely tuned for recognizing facial expressions, leveraging static images from AffectNet alongside a specially devised optimization technique for robustness. The study demonstrates the efficacy of these facial features in swiftly predicting students' engagement levels, their emotional states, and the overall effect of the group. Importantly, this model facilitates real-time video processing directly on each student's mobile device, eliminating the necessity of transmitting facial video data to remote servers or the teacher's PC.

## 3 Proposed framework

**Figure 1:**
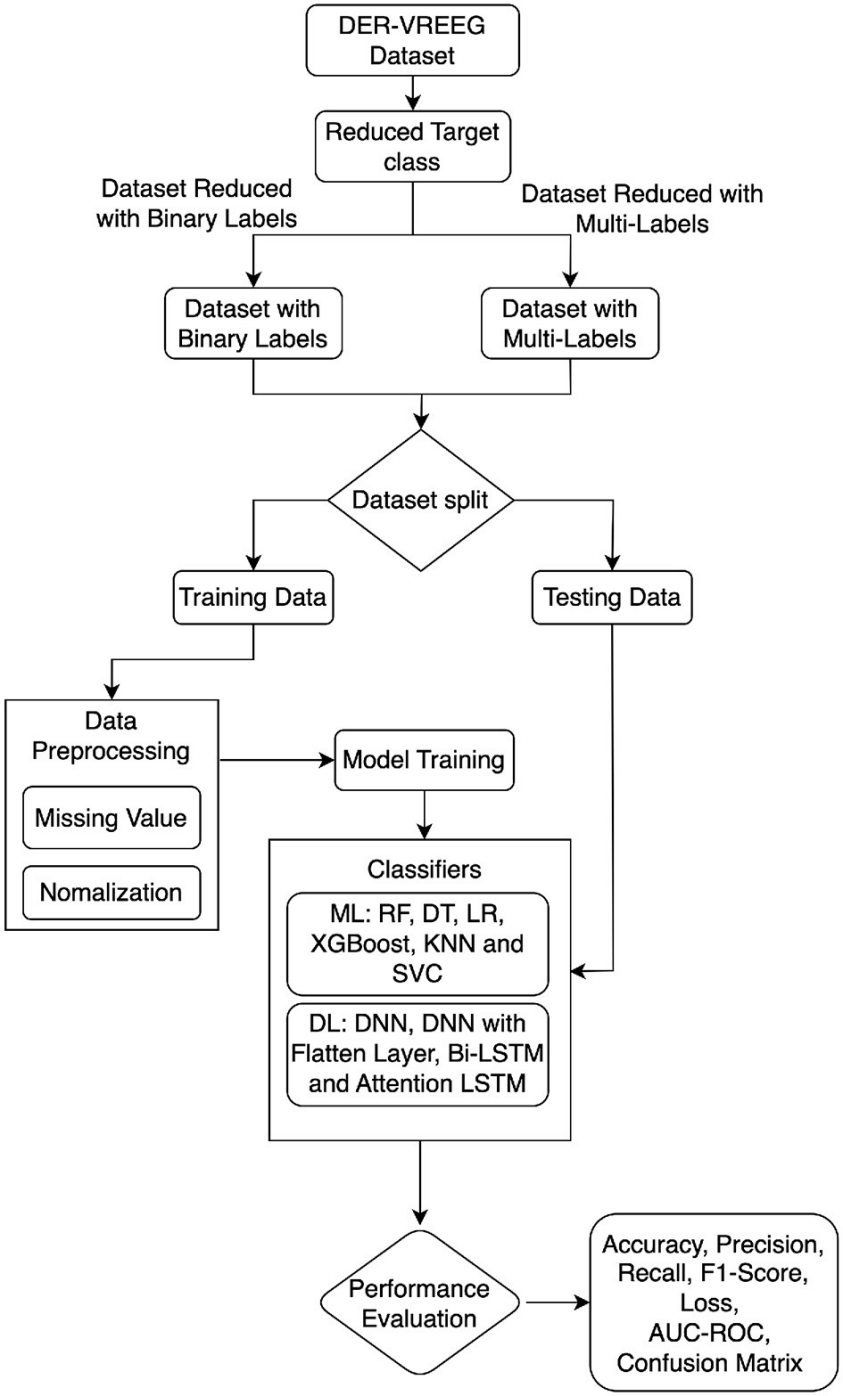
Proposed approach for emotion recognition in virtual reality environment.

This section provides the details of the implementation of the proposed approach using various methodologies and metrics for performance evaluation, including data preliminaries and preprocessing. Figure 1 illustrates the entire process employed in this study for emotional recognition in virtual reality. We utilized the VREED (VR Eyes: Emotions Dataset), a multimodal affective dataset for emotional recognition in virtual reality. Machine learning and deep learning models are applied to the preprocessed dataset.

### 3.1 Dataset preliminaries

**Dataset description:** The dataset used in this study is the VREED (VR Eyes: Emotions Dataset), a multimodal affective dataset sourced from 360 video-based virtual environments (360-VEs) provided via VR headsets. It includes recordings of eye tracking, ECG, Galvanic Skin Reaction (GSR), and self-reported responses from 34 healthy individuals. Participants experienced 360-VEs for one to three minutes each, contributing to the dataset's diverse physiological and behavioral signals.

**Dataset size and source:** VREED consists of 50 features, encompassing a variety of data types aimed at identifying emotions. The dataset is publicly available on Kaggle, facilitating accessibility and reproducibility for researchers (21).

**Features of different classes:** The dataset consists of 50 features in which one feature, Quad_Cat, is a target feature that has four classes. In this research, we implement this dataset with 2 classes and 4 classes to check the performance of the model. After uploading the data, firstly, we used a .replace() method to change the values in the Quad_Cat column. Specifically, all occurrences of 1 are replaced with 0, and all occurrences of 2 and 3 are replaced with 1. After that, correlated variables are dropped to reduce the number of features in the dataset. Dataset features are reduced to 44 features after removing correlated variables.

### 3.2 Dataset preprocessing

Preparing unprocessed data for analysis or modeling is known as data preparation. To properly transform and clean the data for statistical analysis, machine learning, and other data-driven tasks, a number of processes must be taken. This can enhance the efficacy and precision of other data-driven algorithms, such as machine learning models. Several preprocessing techniques were used in this study to modify, standardize, and identify the key data features. Data scientists can make sure that the data is clear, consistent, and formatted correctly for analysis or modeling by carrying out these pretreatment procedures.

The dataset is subjected to various modifications for individual columns using the “ColumnTransformer” class. This is especially helpful if you wish to apply distinct preprocessing processes to each type of data, such as when you have a combination of numerical and categorical features. Next, we applied a “pipeline” class a set of actions sequentially applied to your data-to the data. Typically, an estimator (such as a machine learning model) or a transformation (such as scaling or imputation) make up each pipeline stage.

In our study, rigorous data preprocessing was implemented to ensure the credibility of our proposed method. Initially, missing values in the dataset were identified and handled using appropriate techniques such as imputation or deletion based on the extent and context of missingness. Following this, a Z-score normalization process was applied to standardize the scale and distribution of features. This involved computing the mean and standard deviation for each feature across the dataset and transforming each feature to have a mean of zero and a standard deviation of one. Additionally, feature scaling techniques were employed to adjust the range of values to a uniform scale, further enhancing model convergence and performance. These steps were crucial in preparing the data for robust modeling and ensuring consistent and reliable results across experiments.

Pipelines guarantee that the stages involved in data preprocessing and model training are systematically carried out, which facilitates the development and implementation of machine learning workflows. Next, we imputed missing values from the dataset using a “SimpleImputer” class. It makes it possible to substitute a given strategy like the mean, median, or most frequent value for any missing values in the data. The “StandardScaler” class, a preprocessing transformer that standardizes features by eliminating the mean and scaling them to unit variance, was the last thing we added. This is frequently required for machine learning methods like SVMs and k-means clustering, which are sensitive to the size of the features.

The machine learning technique known as SHAP (SHapley Additive Explanations) is used to comprehend and analyze the predictions made by models. It is predicated on the theory of cooperative games, more precisely on the idea of Shapley values, which were first presented in order to divide rewards among participants in cooperative games equitably. SHAP provides a way to attribute an ensemble model's prediction to its variety of input features within the context of machine learning. It takes into account all potential feature combinations and their effects on the forecast to determine each feature's contribution to the final prediction. This enables a more sophisticated comprehension of the ways in which every attribute influences the model's decision-making procedure.

**Figure 2:**
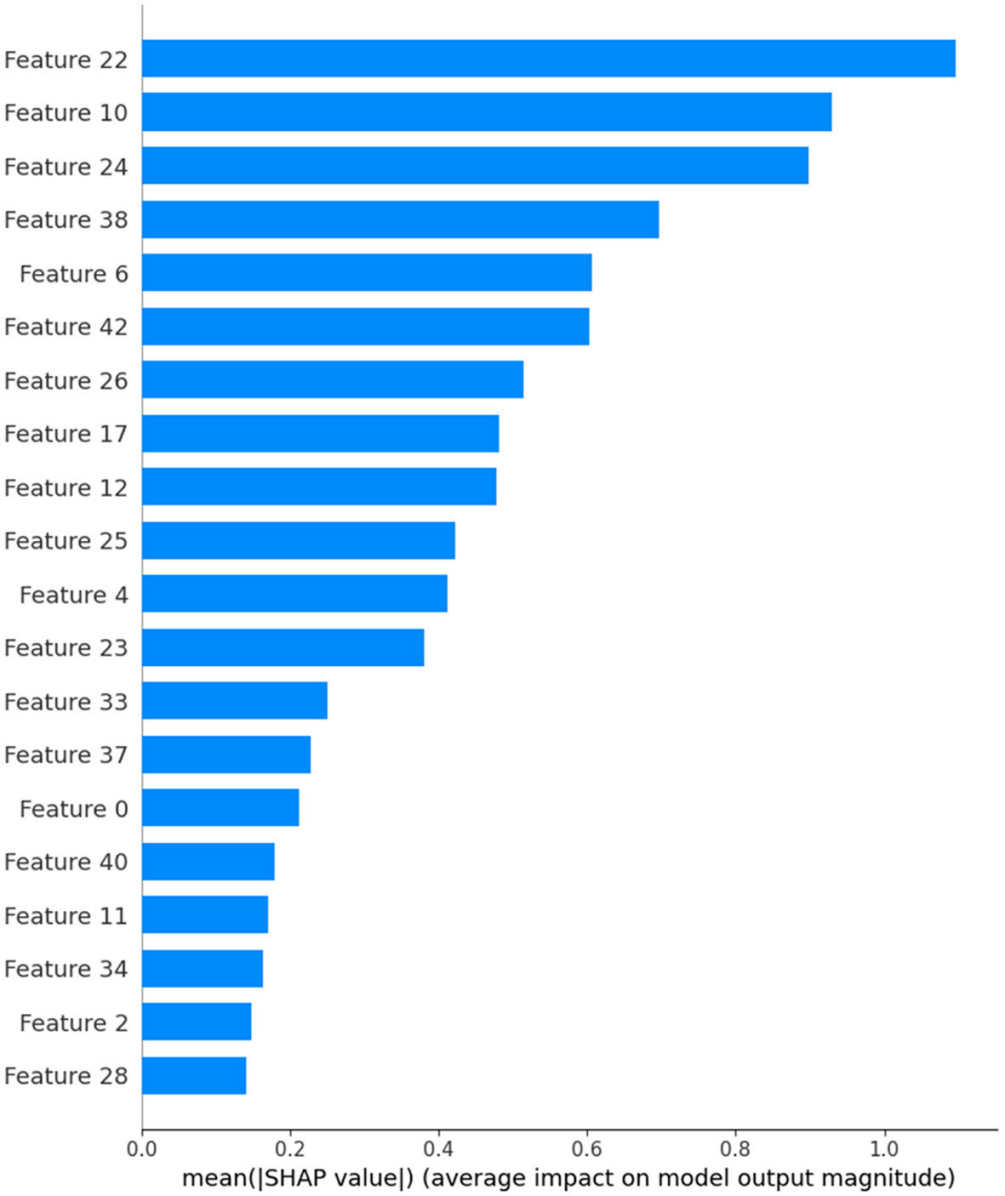
Selected features in binary label dataset.

**Figure 3:**
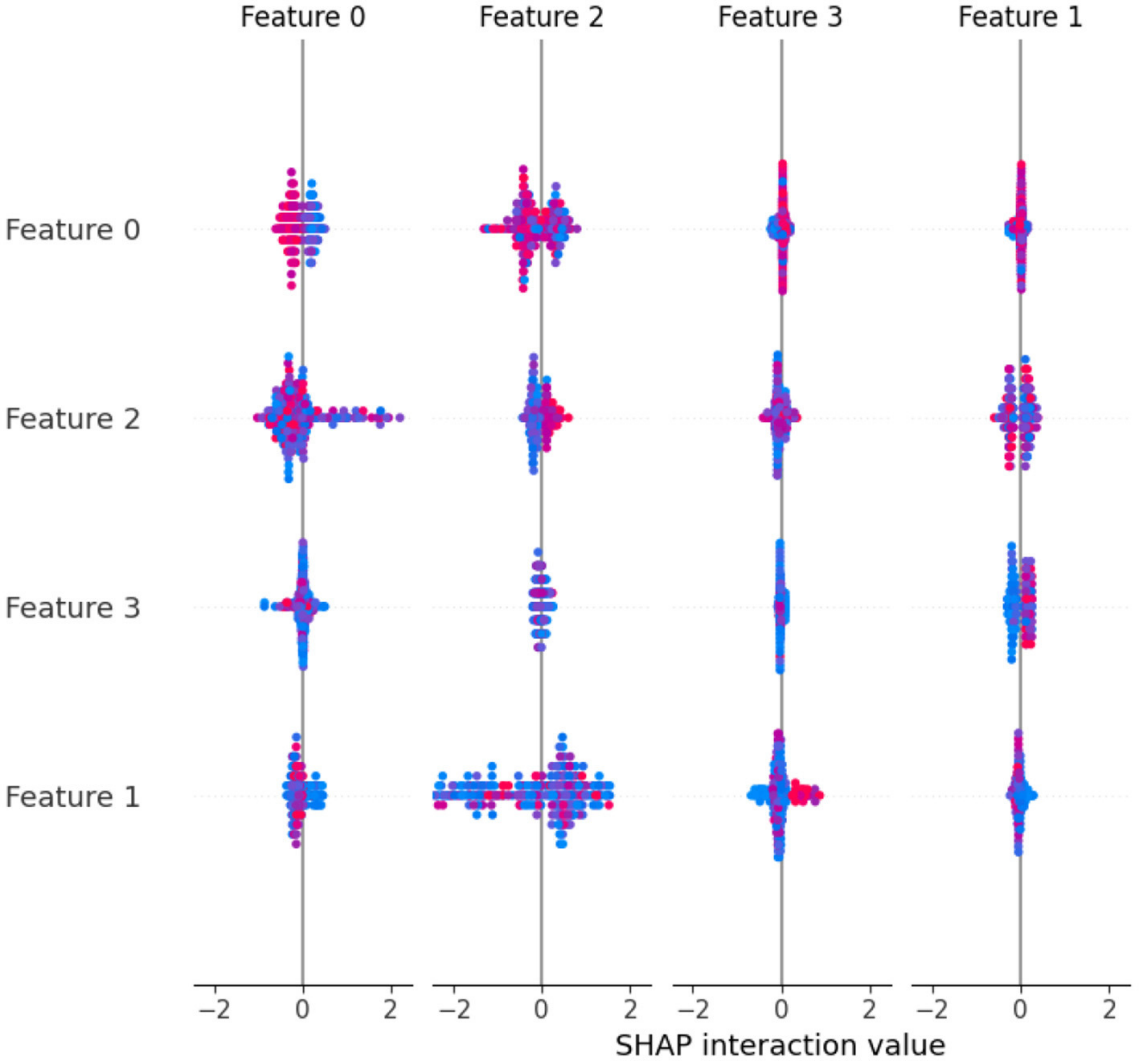
Selected feature in multi-label dataset.

SHAP values shed light on the relative relevance of various traits as well as how they interact. This can be essential to comprehending intricate models like deep neural networks. Practitioners can uncover potential biases, improve model interpretability, and obtain important insights into their models by knowing which features contribute favorably or unfavorably to a prediction. The predictions of the model are explained by the SHAP “TreeExplainer” class. Calling the shap_values method on the TreeExplainer object creates the shap_values variable. The feature importance of the model is then visualized using the summary_plot function from SHAP (Figures 2, 3). The importance of each feature is visualized using a SHAP summary plot, and the mean absolute SHAP values are calculated for each feature. selected_features are these 28, 2, 34, 11, 40, 0, 37, 33, 23, 4, 25, 12, 17, 26, 42, 6, 38, 24, 10, and 22. After feature selection, 20% of the data are used for the testing, and the remaining 80% are used for the training. The random_state parameter is used to initialize the internal random number generator. By setting it to a fixed value (in this case, 42), we ensure that the same random split is generated each time the code is run, which makes the results reproducible.

### 3.3 Classification models

Algorithms that are trained to classify data points into one or more predetermined groups or classes according to their features are called machine learning classifiers. This research used LR, KNN, SVC, DNN, Deep flattened layers, DNN, Bi-LSTM, and classifiers for the evaluation.

**Deep neural network:** Multi-layered hidden layers sit between the input and output layers of deep neural networks (DNNs), which are general-purpose neural networks (22). The last layer's employment of the sigmoid and softmax activation functions indicates that this research employed the model for binary and multi-classification. Following an input layer with 256 nodes, the model consists of three hidden layers of 128, 64, and 64 nodes. With the exception of the final layer, which employs the sigmoid activation function and softmax function for multi-classification, each hidden layer uses the rectified linear unit (ReLU) activation function. To avoid overfitting, dropout regularization is used after each hidden layer, with a dropout rate of 0.3. Additionally, batch normalization is used following every hidden layer to enhance the stability and performance of the model. The binary cross-entropy loss function, accuracy as the evaluation metric, and a learning rate of 0.001 are all features of the Adam optimizer used in the compilation of the model.

**Deep flatten layers DNN:** For classification tasks like image classification, a flattened layer in deep learning reshapes the output of convolutional layers (usually multidimensional arrays) into a 1-dimensional vector, making the transition to fully connected layers easier (23). The sigmoid and softmax activation functions included in the final layer suggest that binary and multi-classification are the model's intended uses. The amount of features in the array determines the form of an input layer in the model. Next, two distinct branches of dense layers with 128 nodes apiece and ReLU activation functions are coupled to the input layer. Following concatenation, the outputs of the two branches are routed via a fully connected layer, including 32 nodes and a ReLU activation function. After being flattened, the output of this layer is routed through a second fully connected layer that has 128 nodes and a ReLU activation function. Lastly, the output layer for binary classification and multi-classification softmax activation function is added. It has a single node and a sigmoid activation function. The model is compiled using the Adam optimizer with a learning rate of 0.001, the evaluation measure being accuracy, and the binary cross-entropy loss function for binary classes and categorical cross-entropy for multi-classes.

**Bi-LSTM:** Bidirectional Long Short-Term Memory is referred to as Bi-LSTM. This kind of Recurrent Neural Network (RNN) architecture simultaneously processes input sequences forward and backward. Thus, by collecting relationships and patterns in the input sequence from both past and future contexts, the network can interpret and represent sequential data more effectively (24). The architecture of the Bi-LSTM model consists of three stacked Bi-LSTM layers, each consisting of 128, 64, and 32 units. A dropout layer is positioned after the layers to prevent overfitting. The binary cross-entropy loss function and Adam optimizer are used to create the model.

**Attention LSTM:** An attention mechanism and the Long Short-Term Memory (LSTM) architecture are coupled to form an Attention LSTM, or LSTM with Attention Mechanism. An Attention LSTM enables the model to dynamically pay to distinct portions of the input sequence at each time step as the attention mechanism is embedded into the LSTM architecture (25). This makes it possible for the model to represent dependencies better and generate predictions, particularly in situations where specific input sequence elements are more crucial than others. In this work, we integrate an attention mechanism for representation learning with the LSTM and GRU layers, and then we proceed to fully linked layers for transfer learning and self-supervised learning. A 64-unit LSTM layer is defined, and its result is passed through an attention mechanism. The attention mechanism returns a weighted sum of the LSTM output after receiving both itself and the LSTM output as inputs. The output of a 16-unit GRU layer is defined and concatenated with the attention output. There are two completely connected layers defined, one for the GRU output and one for the attention output, both with sixteen units. The TimeDistributed wrapper is used to apply these layers, applying the same layer to the input sequence at each time step. The flattened layer is used to flatten the output of the fully linked layers, and the concatenate layer is used to concatenate the two flattened outputs. For binary classification, a final output layer with a single unit and sigmoid activation is defined after a fully connected layer with 64 units with ReLU activation. The binary cross-entropy for binary classes, categorical cross-entropy for multi-class classes, and the Adam optimizer are used in the compilation of the model.

Algorithm 1 describes an Emotional Recognition system in Virtual Reality (ER-VREEG) that makes use of both ML and DL classifiers. The ER-VREEG dataset is the input, while the Emotional Recognition in Virtual Reality (*E*_*R*_) dataset is the output. The first function takes in the ER-VREEG dataset and returns a reduced dataset with binary and multi-label targets. The second function normalizes the data and deals with missing values. It returns the preprocessed data after receiving the reduced dataset as input. The third function defines several machine learning classifiers, including SVC, KNN, and LR. It returns the ML classifiers after training. Bidirectional Long Short-Term Memory (Bi-LSTM), Attention Long Short-Term Memory (attention LSTM), DNN, and DNN with flattened layers are among the DL classifiers defined by the third function. The trained DL classifier is returned. The function finally returns the ML and DL classifiers' performance.

**Algorithm 1:**
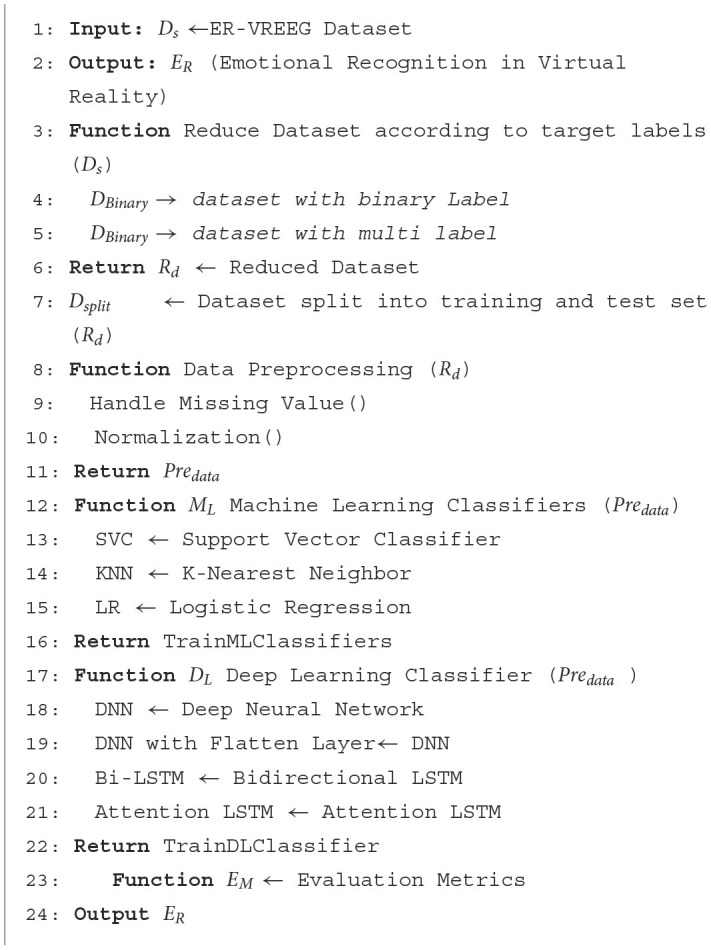
Pseudo code for emotional recognition in virtual reality environment.

## 4 Experimental result and discussion

This section presents the experimental analysis, results and discussion of the proposed approach. A model for virtual reality emotion recognition that integrates ML and DL classifiers is presented. It uses 20% of the dataset for testing and 80% of the dataset for training. Performance is evaluated using predetermined metrics, including F1-score, accuracy, precision, recall, and loss. For effective development, Jupyter Notebook and Evaluation criteria such as accuracy, precision, recall, and F1-score were employed to assess the efficacy of the model. Each of the mathematical formulae for the prediction and classification problems is given below. The assessment measurements are used to evaluate the problems. The accuracy of the classifier is used to determine its overall correctness. Out of all the cases, it shows the proportion of cases that were successfully classified. The accuracy of the classifier can be assessed by dividing the total number of positive predictions by the proportion of actual positive predictions. Stated differently, it assesses the classifier's ability to avoid producing false positives.

The classification report of a number of machine learning classifiers, such as KNN, SVC, and LR, is displayed in Table 1. Along with the overall accuracy and weighted average, performance parameters of each classifier are provided for each class (0 and 1), including precision, recall, F1-score, and support. For both classes, SVC classifiers perform well. The classifiers for KNN, SVC, and LR all perform well, with F1 scores, recall, and precision ranging from 0.71 to 0.90. High accuracy, weighted average scores, and macro averages are attained by all classifiers, demonstrating strong overall performance over the dataset.

**Table 1:** Classification report of ML classifiers for binary classes.

	**Labels**	**Precision**	**Recall**	**F1- support**	**Support**
LR	0	0.76	0.77	0.76	118
	1	0.79	0.78	0.78	131
	Accuracy	-	-	0.78	249
	Wei. Avg	0.78	0.78	0.78	249
SVC	0	0.86	0.90	0.88	118
	1	0.90	0.87	0.89	131
	Accuracy	-	-	0.88	249
	Wei. Avg	0.88	0.88	0.88	249
K-NN	0	0.71	0.77	0.74	118
	1	0.78	0.72	0.75	131
	Accuracy	-	-	0.74	249
	Wei. Avg	0.75	0.74	0.74	249

The classification report of several DL classifiers, such as Attention LSTM, Bi-LSTM, DNN, and DNN with deep flattened layers, is shown in Table 2. Performance parameters for each classifier, including precision, recall, F1-score, and support, are given for each class (0 and 1) in addition to the overall accuracy and weighted average. Strong performance is demonstrated by all DL classifiers, with excellent recall, F1 scores, and precision for both classes. An F1-score of 0.90 is obtained by DNN and DNN with deep flattening layers, suggesting equal performance across precision and recall for both classes. The Bi-LSTM effectively captures long-range relationships in the input sequence, as evidenced by its F1-score of 0.90. For both classes, Attention LSTM receives perfect scores (1.00) for every metric, demonstrating remarkable performance and the capacity to choose and focus on pertinent segments of the input sequence.

**Table 2:** Classification report of DL classifiers for binary classes.

	**Labels**	**Precision**	**Recall**	**F1- support**	**Support**
DNN	C0	0.90	0.90	0.90	118
	C1	0.91	0.91	0.91	131
	Accuracy	-	-	0.90	249
	Wei. Avg	0.90	0.90	0.90	249
Deep flatten layers DNN	C0	0.93	0.97	0.95	118
	C1	0.97	0.93	0.95	131
	Accuracy	-	-	0.95	249
	Wei. Avg	0.95	0.95	0.95	249
Bi-Lstm	C0	0.85	0.95	0.90	118
	C1	0.95	0.85	0.90	131
	Accuracy	-	-	0.90	249
	Wei. Avg	0.90	0.90	0.90	249
Attention LSTM	C0	1.00	1.00	1.00	113
	C1	1.00	1.00	1.00	118
	Accuracy	-	-	1.00	249
	Wei. Avg	1.00	1.00	1.00	249

Table 3 the classification report for multi-class of all ML classifiers for each class (0 and 1), as well as for the overall accuracy and weighted average. LR, SVC and K-NN classifiers exhibit good performance, with precision, recall, and F1-score ranging from 0.71 to 1.00. All classifiers achieve high accuracy and weighted average scores, indicating robust overall performance across the dataset.

**Table 3:** Classification report of ML classifiers for multi-classes.

	**Labels**	**Precision**	**Recall**	**F1- support**	**Support**
LR	0	0.76	0.77	0.76	118
	1	0.79	0.78	0.78	131
	Accuracy	-	-	0.78	249
	Wei. Avg	0.78	0.78	0.78	249
SVC	0	0.86	0.90	0.88	118
	1	0.90	0.87	0.89	131
	Accuracy	-	-	0.88	249
	Avg	0.88	0.88	0.88	249
K-NN	0	0.71	0.77	0.74	118
	1	0.78	0.72	0.75	131
	Accuracy	-	-	0.74	249
	Wei. Avg	0.75	0.74	0.74	249

Key: Weighted-Wei.

Table 4 the classification report for all DL classifiers in a multi-class setting for each class (0, 1, 2, and 3). DNN achieves perfect precision, recall, and F1-score (1.00) for all classes, indicating excellent performance. In all classes, with minor deviations, Deep Flatten Layers DNN shows good precision, recall, and F1-score. With a weighted average F1-score of 0.93, the total accuracy increases to 0.93. While significantly less than other models, Bi-LSTM performs well with excellent recall, precision, and F1-score. With a weighted average F1-score of 0.89, the total accuracy is 0.89. For every lesson, Attention LSTM attains flawless precision, recall, and F1-score (1.00), signifying remarkable performance. With a weighted average F1-score of 1.00, the overall accuracy is 1.00.

**Table 4:** Classification report of DL classifiers for multi-classes.

	**Labels**	**Precision**	**Recall**	**F1- support**	**Support**
DNN	C0	1.00	1.00	1.00	63
	C1	1.00	1.00	1.00	55
	C2	1.00	1.00	1.00	65
	C3	1.00	1.00	1.00	66
	Accuracy	-	-	0.81	249
	Wei. Avg	1.00	0.81	0.81	249
Deep flatten layers DNN	C0	0.91	0.98	0.95	63
	C1	0.88	0.91	0.89	55
	C2	0.92	0.89	0.91	65
	C3	1.00	0.92	0.96	66
	Accuracy	-	-	0.93	249
	Wei. Avg	0.93	0.93	0.93	249
Bi-LSTM	C0	0.87	0.95	0.91	63
	C1	0.82	0.84	0.83	55
	C2	0.89	0.85	0.87	65
	C3	0.98	0.92	0.95	66
	Accuracy	-	-	0.89	249
	Wei. Avg	0.89	0.89	0.89	249
Attention LSTM	C0	1.00	1.00	1.00	63
	C1	1.00	1.00	1.00	55
	C2	1.00	1.00	1.00	65
	C3	1.00	1.00	1.00	66
	Accuracy	-	-	1.00	249
	Wei. Avg	1.00	1.00	1.00	249

**Figure 4:**
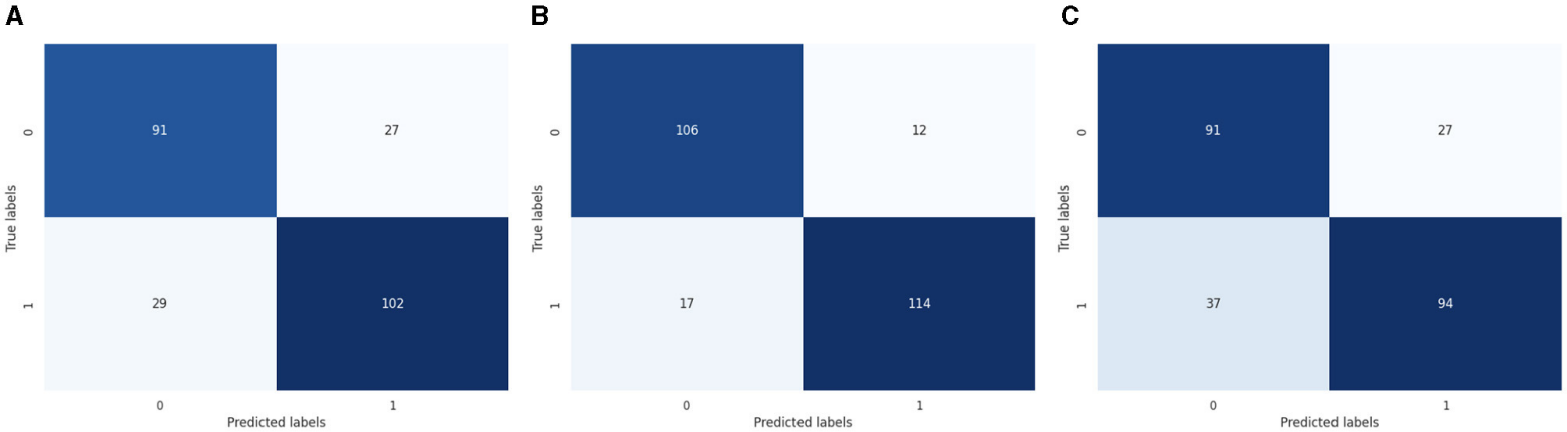
ML-CM for binary classes. **(A)** CM of logistic regression. **(B)** CM of SVC. **(C)** CM of KNN.

The machine learning model for the binary class is shown graphically in Figure 4. CM of the LR model, which is shown in Figure 4; the model performed well, as seen by the greater value of TP, where the true and predicted label was 0 with a value of 91, and TN, where the true and predicted label was 1, with a value of 102. In the case of FP, where the real label was 1, and the predicted label was 0, with a value of 29, and FN, where the true label was 0, and the expected label was 1, with a value of 27, the LR model incorrectly predicted a lower value. CM of the SVC model, the model performed well, as seen by the greater value of TP, where the true and predicted label was 0 with a value of 106, and TN, where the true and predicted label was 1, with a value of 114. In the case of FP, where the real label was 1, and the predicted label was 0, with a value of 17, and FN, where the true label was 0, and the expected label was 1, with a value of 12, the LR model incorrectly predicted a lower value. CM of the K-Nearest Neighbors model, the model performed well, as seen by the greater value of TP, where the true and predicted label was 0 with a value of 91, and TN, where the true and predicted label was 1, with a value of 94. In the case of FP, where the real label was 1, and the predicted label was 0, with a value of 37, and FN, where the true label was 0, and the expected label was 1, with a value of 27, the LR model incorrectly predicted a lower value.

**Figure 5:**
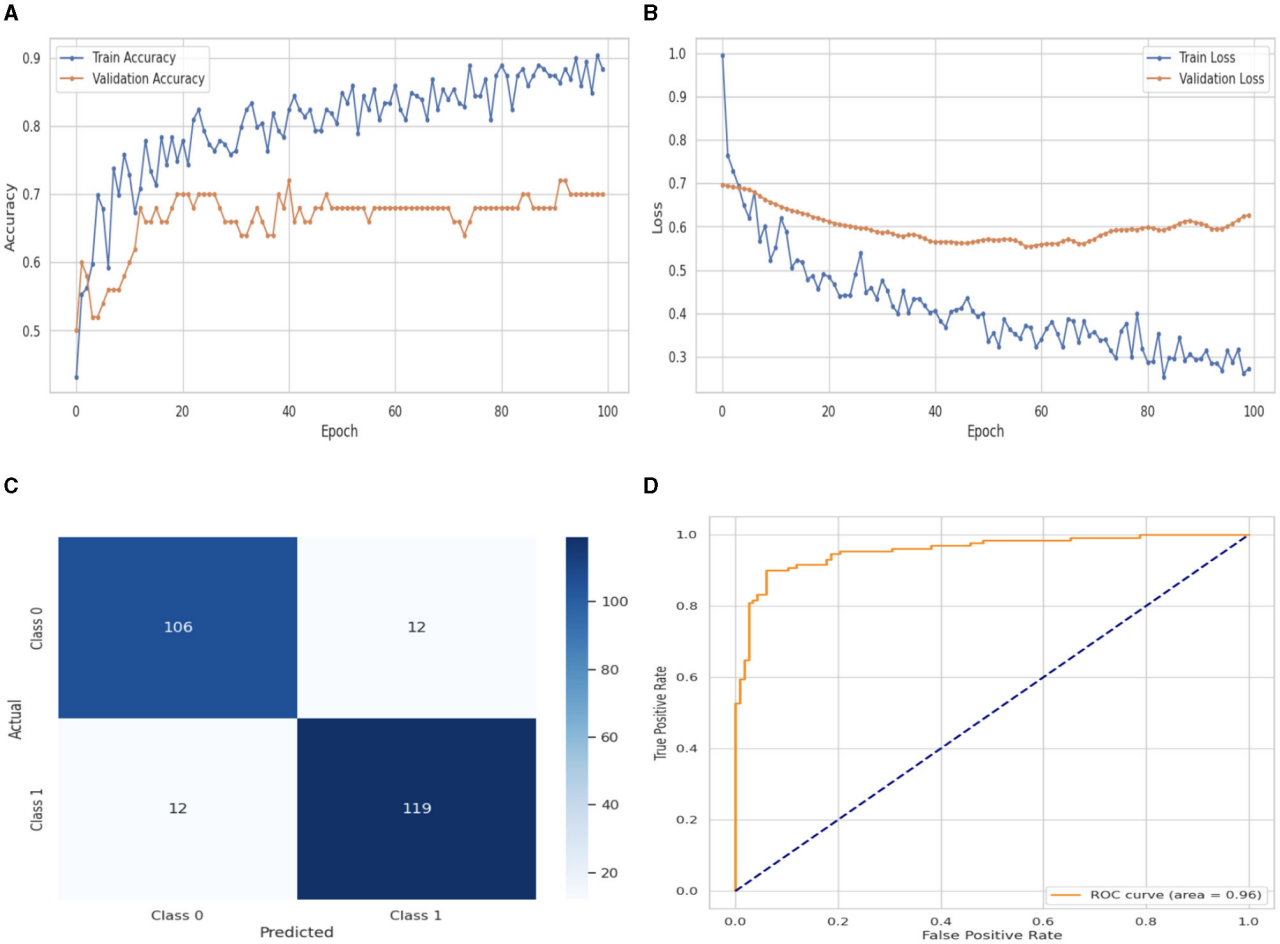
Performance visualization of DNN model. **(A)** Train accuracy. **(B)** Train loss. **(C)** CM. **(D)** ROC.

The DNN classifier's performance evaluation is shown in Figure 5, which includes training and validation loss and accuracy. Figure 5A displays the accuracy graph for training and validation curves. The DNN classifier's training accuracy begins at 0_*th*_ epoch and is 0.40%. This study evaluates the result in 100 epochs, and after some fluctuation of increases and decreases, the last value we get is 0.89% at 100_*th*_ epoch. The validation accuracy starts at 0_*th*_ epoch with a value of 0.50% and after some fluctuation of increases, decreases, and sometimes remains constant value, then at last value we get 0.70% at 100_*th*_ epoch. The training and validation loss graph is shown in Figure 5B. The training loss of the DNN classifier starts at 0_*th*_ epoch with a value of 1.0. It decreases up to 0.27. at 100_*th*_ epoch, and the validation loss starts at 0_*th*_ epoch with a value of 0.7 and decreases up to 0.65 at 100_*th*_ epoch. CM of the DNN model, which is shown in Figure 5C, the model performed well, as seen by the greater value of TP, where the true and predicted label was 0 with a value of 106, and TN, where the true and predicted label was 1, with a value of 119. The lower values of FP and FN, where the real label was 0 and the predicted label was 1 with a value of 12, respectively, were incorrectly predicted by the DNN model. The ROC curve illustrates the model's performance at various classification thresholds. A True Positive Rate (TPR) or Sensitivity graph is plotted on the y-axis, while an x-axis indicates the False Positive Rate (FPR). Indicating how successfully the model discriminates across classes, the curve displays the trade-off between sensitivity and specificity. It is better if the performance curve is closer to the upper-left corner. The orange line indicates the performance of the DNN model in the ROC curve for binary class with value 0.96% in Figure 5D.

**Figure 6:**
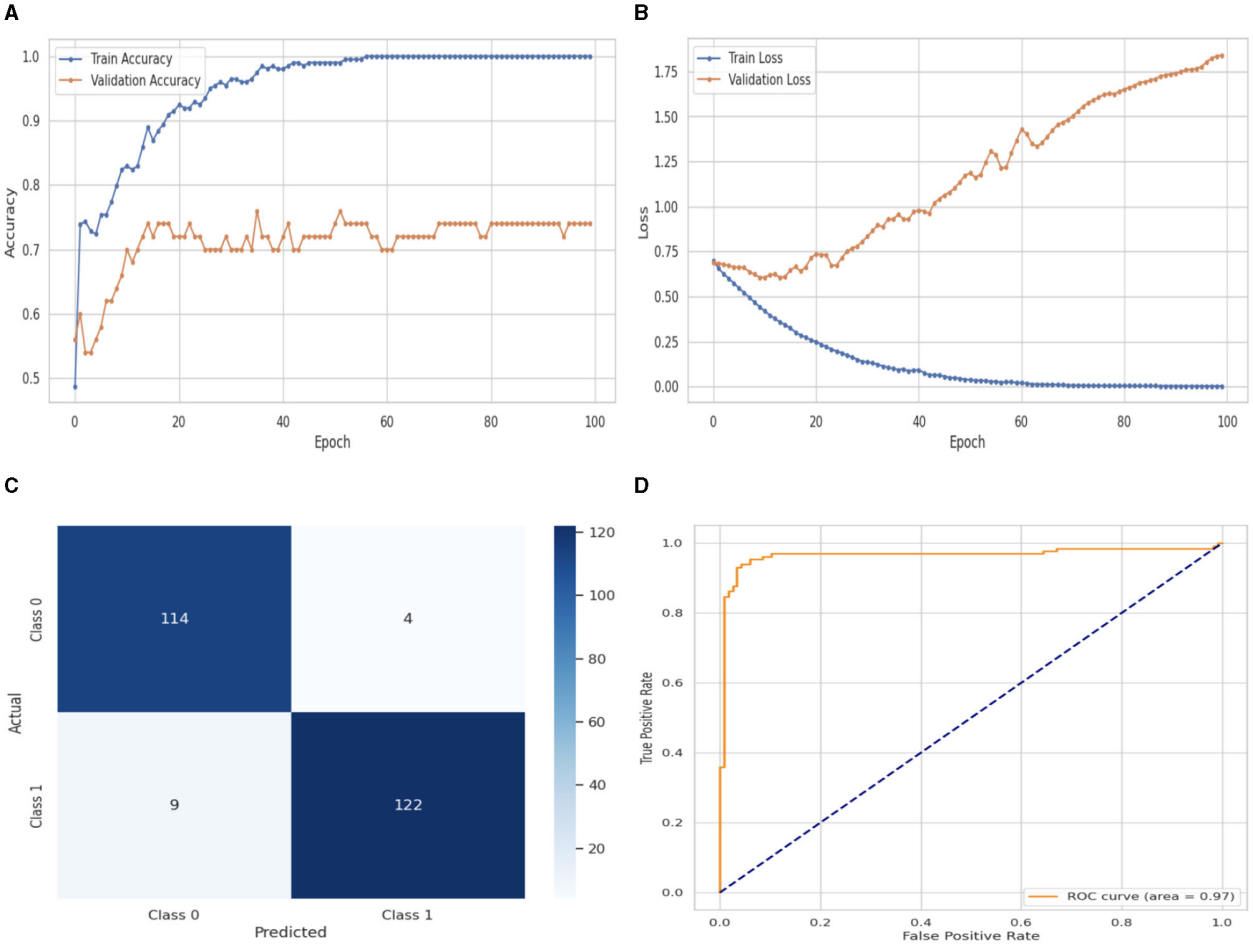
Performance visualization of DNN with flatten layer. **(A)** Train accuracy. **(B)** Train loss. **(C)** CM. **(D)** ROC.

**Figure 7:**
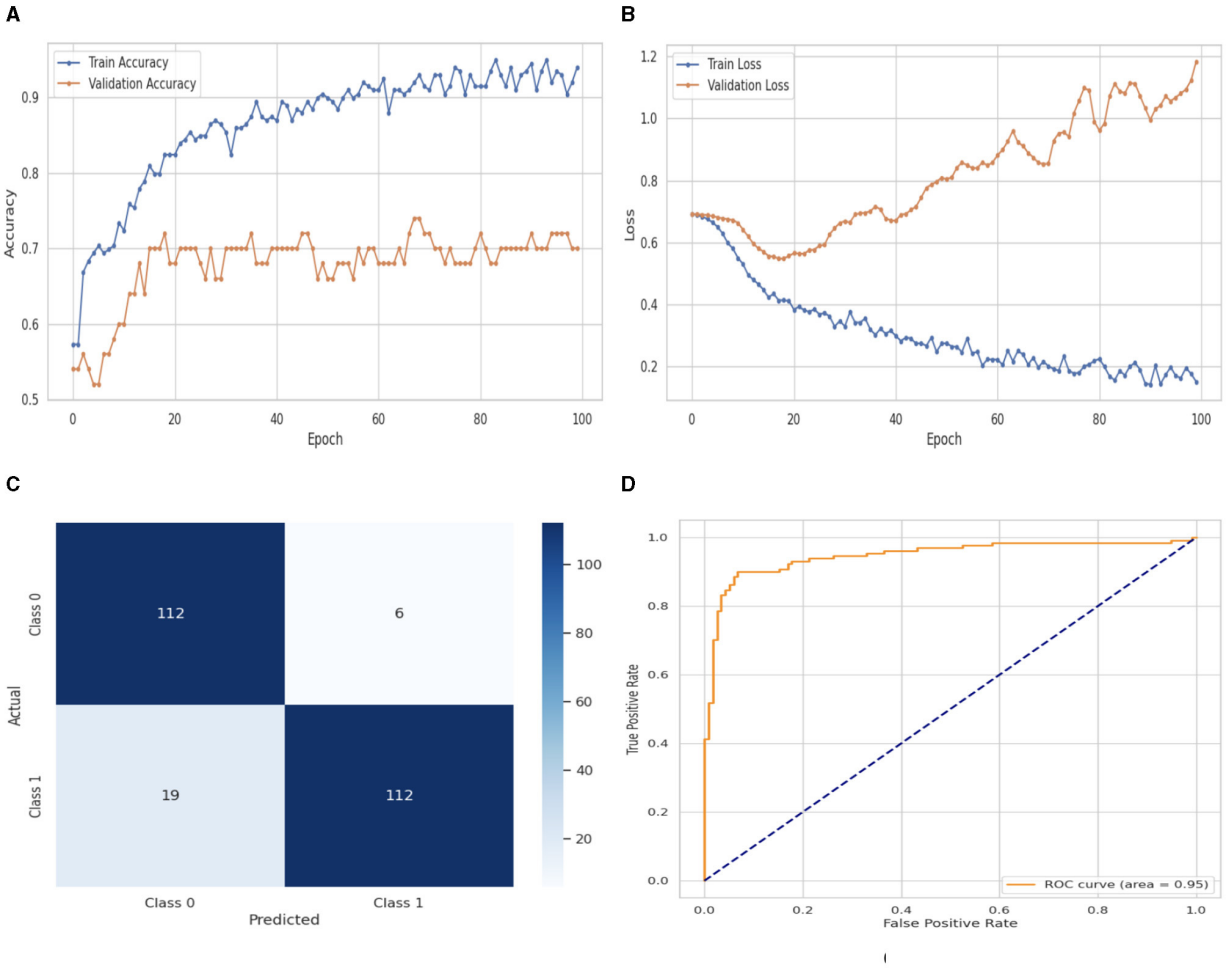
Performance visualization of Bi-LSTM. **(A)** Train accuracy. **(B)** Train loss. **(C)** CM. **(D)** ROC.

Figure 6 illustrates the performance evaluation of the DNN with a flattened layer with training and validation loss and accuracy. Figure 6A presents the training and validation accuracy graphs. The training accuracy of the DNN classifier starts at 0_*th*_ epoch with a value of 0.48%. This study evaluates the result in 100 epochs, and after some fluctuation of increases and decreases, the last value we get is 100% at 100_*th*_ epoch. The validation accuracy starts at 0_*th*_ epoch with a value of 0.56% and after some fluctuation of increases, decreases, and sometimes remains constant value, then at the last value we get 0.74% at 100_*th*_ epoch. Figure 6B presents the training and validation loss graph. The training loss of the DNN with flattened layer classifier starts at 0_*th*_ epoch with a value of 0.71. It decreases up to 0.01. at 100_*th*_ epoch, and the validation loss starts at 0_*th*_ epoch with a value of 0.71 and decreases up to 1.78 at 100_*th*_ epoch. CM of the DNN model with flattened layer, which is shown in Figure 7C, the model performed well, as seen by the greater value of TP, where the true and predicted label was 0 with a value of 114, and TN, where the true and predicted label was 1, with a value of 122. The lower values of FP and FN, where the real label was 0 and the predicted label was 1 with a value of 4, respectively, were incorrectly predicted by the DNN model. A TPR or sensitivity graph is plotted on the y-axis, while an x-axis indicates the FPR. Indicating how successfully the model discriminates across classes, the curve displays the trade-off between sensitivity and specificity. It is better if the performance curve is closer to the upper-left corner. The orange line indicates the performance of the DNN model with a flattened layer in the ROC curve for binary class with value 0.97% in Figure 6D.

Figure 7 illustrates the performance evaluation of the Bi-LSTM with training and validation loss and accuracy. In Figure 7A, the training accuracy of the Bi-LSTM classifier starts at 0_*th*_ epoch with a value of 0.58%. This study evaluates the result in 100 epochs, and after some fluctuation of increases and decreases, the last value we get is 0.98% at 100_*th*_ epoch. The validation accuracy starts at 0_*th*_ epoch with a value of 0.53% and after some fluctuation of increases, decreases, and sometimes remains constant value, then at the last value we get 0.70% at 100_*th*_ epoch. Figure 7B presents the training and validation loss graph. The training loss of the Bi-LSTM classifier starts at 0_*th*_ epoch with a value of 0.7. It decreases up to 0.18. at 100_*th*_ epoch, and the validation loss starts at 0_*th*_ epoch with a value of 0.7 and decreases up to 1.19 at 100_*th*_ epoch. CM of the Bi-LSTM, which is shown in Figure 7C, the model performed well, as seen by the greater value of TP, where the true and predicted label was 0 with a value of 112, and TN, where the true and predicted label was 1, with a value of 112. The lower values of FP and FN, where the real label was 0 and the predicted label was 1 with a value of 6, respectively, were incorrectly predicted by the Bi-LSTM model. Indicating how successfully the model discriminates across classes, the curve displays the trade-off between sensitivity and specificity. It is better if the performance curve is closer to the upper-left corner. The orange line indicates the performance of the Bi-LSTM model in the ROC curve for binary class with value 0.95% in Figure 7D.

**Figure 8:**
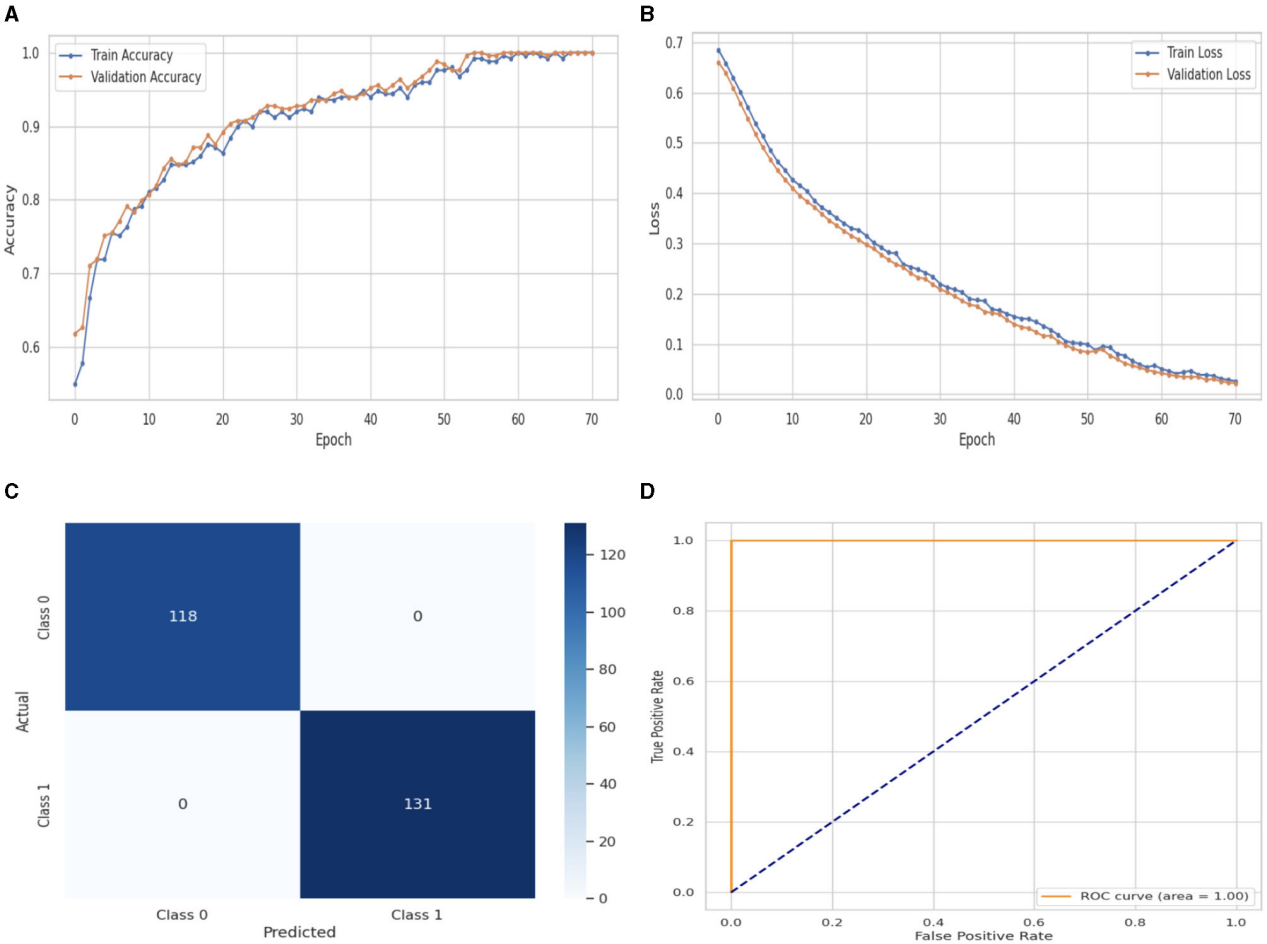
Performance visualization of attention LSTM. **(A)** Train Accuracy. **(B)** Train loss. **(C)** CM. **(D)** ROC.

Figure 8 illustrates the performance evaluation of the Attention LSTM with training and validation loss and accuracy. In Figure 8A, the training accuracy of the Attention LSTM classifier starts at 0_*th*_ epoch with a value of 0.52%. This study evaluates the result in 100 epochs, and after some fluctuation of increases and decreases, the last value we get is 100% at 100_*th*_ epoch. The validation accuracy starts at 0_*th*_ epoch with a value of 0.61% and after some fluctuation of increases, decreases, and sometimes remains constant value, then at the last value we get 100% at 100_*th*_ epoch. Figure 8B presents the training and validation loss graph. The training loss of the Attention LSTM classifier starts at 0_*th*_ epoch with a value of 0.69. It decreases up to 0.01. at 100_*th*_ epoch, and the validation loss starts at 0_*th*_ epoch with a value of 0.68 and decreases up to 0.01 at 100_*th*_ epoch. CM of the Attention LSTM, which is shown in Figure 8C, the model performed well, as seen by the greater value of TP, where the true and predicted label was 0 with a value of 118, and TN, where the true and predicted label was 1, with a value of 131. The lower values of FP and FN, where the real label was 0 and the predicted label was 1 with a value of 0, respectively, were incorrectly predicted by the Attention LSTM model. Indicating how successfully the model discriminates across classes, the curve displays the trade-off between sensitivity and specificity. It is better if the performance curve is closer to the upper-left corner. The orange line indicates the performance of the Attention LSTM model in the ROC curve for binary class with value 100% in Figure 8D.

**Figure 9:**
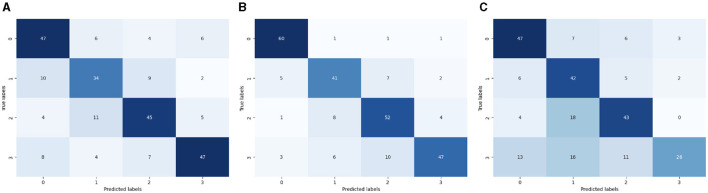
ML-CM of multi-classes. **(A)** CM of logistic regression. **(B)** CM of SVC. **(C)** CM of KNN.

Figure 9 shows the confusion matrix of the ML multi-class label. The diagonal cells of the matrix show the number of occurrences correctly identified, and the off-diagonal cells show the proportion of instances that were wrongly classified. Figure 9A presents the CM of LR. The x-axis shows the predicted labels and the y-axis represents the true labels, which are 0, 1, 2, and 3. 47 instances were projected for label 0, 34 for label 1, 45 for label 2 and 47 for label 3 accurately anticipated. Figure 9B presents the CM of SVC. The x-axis shows the predicted labels and the y-axis represents the true labels, which are 0, 1, 2, and 3. 60 instances were projected for label 0, 41 for label 1, 52 for label 2 and 47 for label 3 accurately anticipated. Figure 9C presents the CM of KNN. The x-axis shows the predicted labels and the y-axis represents the true labels, which are 0, 1, 2, and 3. 47 instances were projected for label 0, 42 for label 1, 43 for label 2 and 26 for label 3 accurately anticipated.

**Figure 10:**
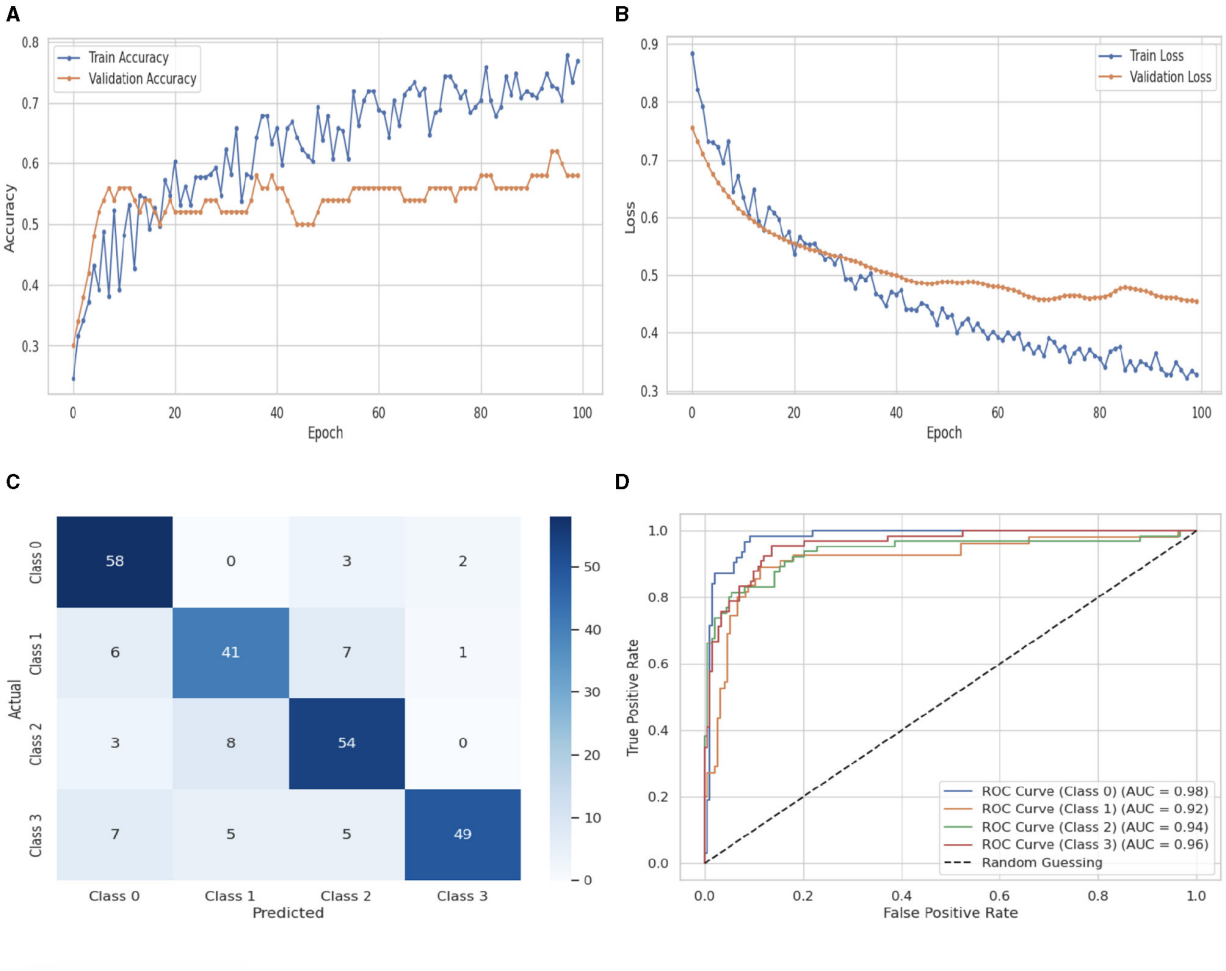
Performance visualization of DNN. **(A)** Train accuracy. **(B)** Train loss. **(C)** CM. **(D)** ROC.

Figure 10 illustrates the performance evaluation of the DNN classifier. Figure 10A presents the training and validation accuracy graph. The training accuracy of the DNN classifier starts at 0_*th*_ epoch with a value of 0.23%. After some fluctuations of increase and decrease. It increases up to 0.78% at 100_*th*_ epoch. The validation accuracy starts at 0_*th*_ epoch with a value of 0.3%, and after some fluctuations increase and decrease and remain constant, it increases up to 0.59% at 100_*th*_ epoch. Figure 10B presents the training and validation loss graph. The training loss of the DNN classifier starts at 0_*th*_ epoch with a value of 0.89. After some fluctuations of increase and decrease. It decreases up to 0.32 at 100_*th*_ epoch, and the validation loss starts at 0_*th*_ epoch with a value of 0.77, and after some fluctuations, it decreases up to 0.46 at 100_*th*_ epoch. Figure 10C shows the confusion matrix of the DNN. 58 instances were projected for label 0, 41 instances for C1, 54 instances for C2, and 49 instances for C3 are accurately anticipated. Indicating how successfully the model discriminates across classes, the curve displays the trade-off between sensitivity and specificity. It is better if the performance curve is closer to the upper-left corner. Here, categorization is done using four classifications. C0 is represented by the orange line, C1 is represented by the green line, C3 is represented by the red line, and C4 represents the blue line. The ROC curve for each class in the LR model is displayed in Figure 10D. The studies are conducted in four classes: C0 scored 0.98%, C1 scored 0.92%, C2 scored 0.94%, and C3 scored 0.96%.

**Figure 11:**
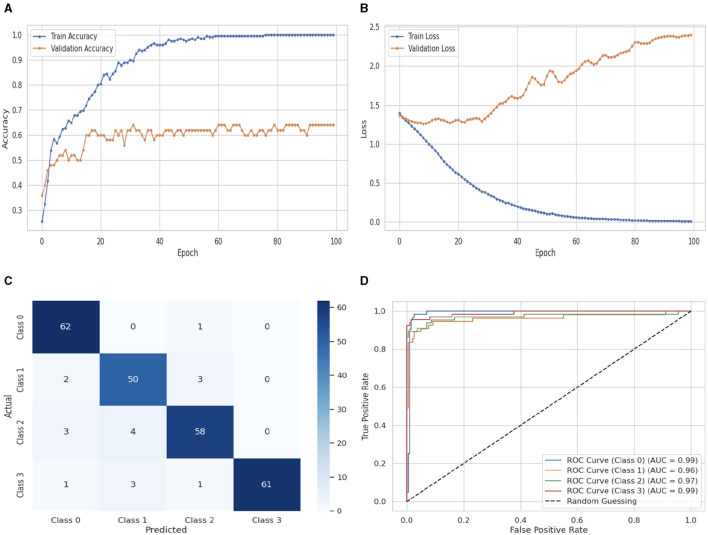
Performance visualization of DNN flatten layer. **(A)** Train accuracy. **(B)** Train loss. **(C)** CM. **(D)** ROC.

Figure 11 illustrates the performance evaluation of the DNN with a flattened layer classifier. As shown in Figure 11A, the DNNflatten classifier's training accuracy begins at 0_*ht*_ epoch and has a value of 0.23%. It eventually stays consistent after experiencing some ups and downs. A maximum growth of 1.0% occurs at 100_*th*_ epoch. The validation accuracy has a value of 0.36% at 0_*th*_ epoch. After some variations that rise, fall, and stay constant, it rises to 0.53% at 100_*th*_ epoch. The training and validation loss graph is shown in Figure 11B. The training loss of the DNNflatten classifier starts at 0_*th*_ epoch with a value of 1.4, it decreases up to 0.0 at 100_*th*_ epoch, and the validation loss starts at 0_*th*_ epoch with a value of 1.4. After some fluctuations, it increases up to 2.4 at 100_*th*_ epoch. Figure 11C shows the confusion matrix of the DNNflatten. Sixty two instances were projected for label 0, 50 instances for C1, 58 instances for C2, and 61 instances for C3 are accurately anticipated. Indicating how successfully the model discriminates across classes, the curve displays the trade-off between sensitivity and specificity. It is better if the performance curve is closer to the upper-left corner. Here, categorization is done using four classifications. The ROC curve for each class of the DNNflatten model is displayed in Figure 11D. The studies are conducted in four classes: C0 scored 0.99%, C1 scored 0.96%, C2 scored 0.97%, and C3 scored 0.99%.

**Figure 12:**
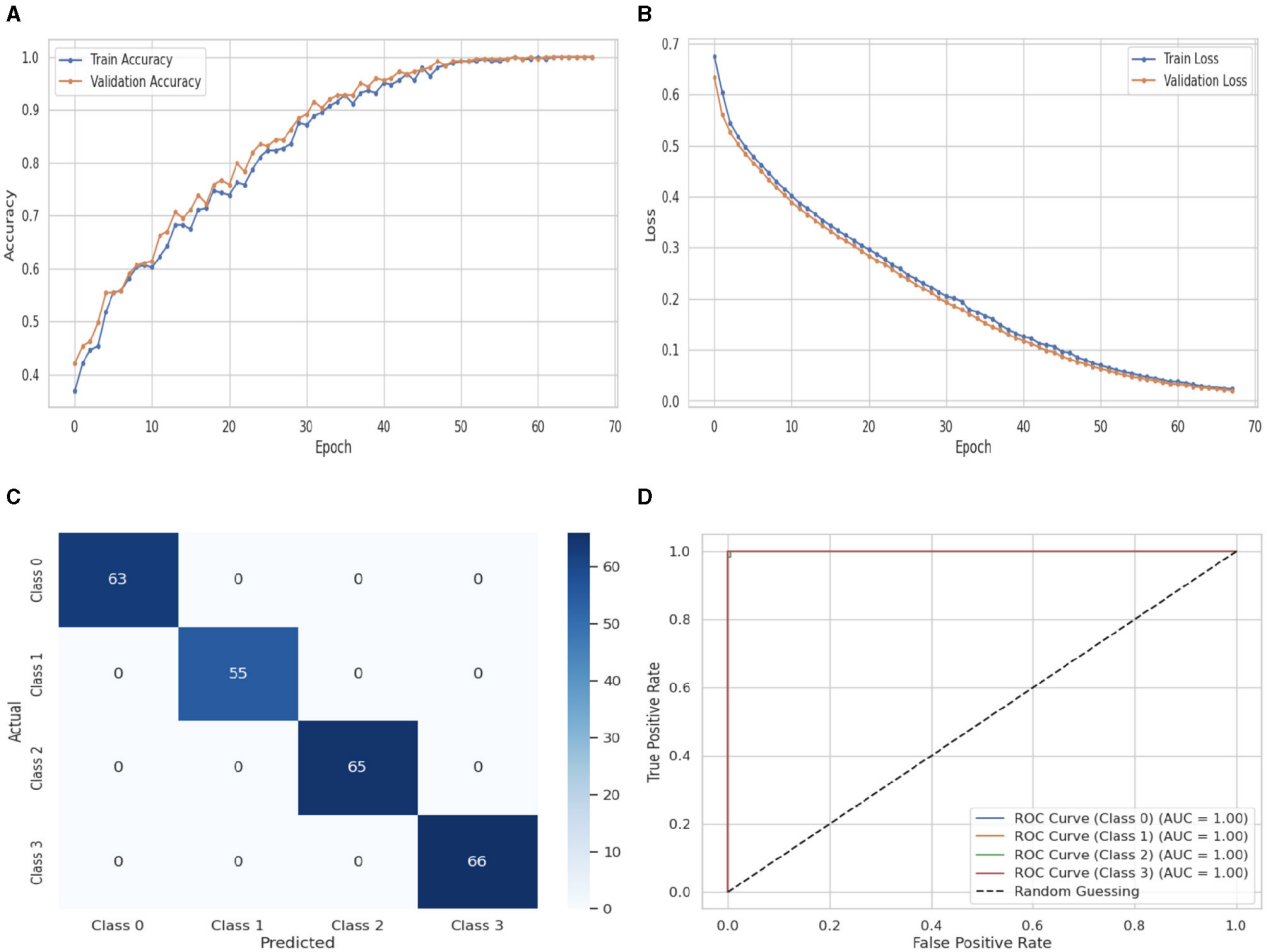
Performance visualization of Bi-LSTM. **(A)** Train accuracy. **(B)** Train loss. **(C)** CM. **(D)** ROC.

Figure 12 illustrates the performance evaluation of the Bi-LSTM classifier. In Figure 12A, the training accuracy of the Bi-LSTM classifier starts at 0_*th*_ epoch with a value of 0.2%. After some fluctuations increase and decrease, it increases up to 1.0% at 170_*th*_ epoch and the validation accuracy starts at 0_*st*_ epoch with a value of 0.42%. After some fluctuations increase and decrease and remain constant, it increases up to 1.0% at 70_*th*_ epoch. Figure 12B presents the training and validation loss graph. The training loss of the Bi-LSTM classifier starts at 0_*th*_ epoch with a value of 0.7. It decreases up to 0.0 at 70_*th*_ epoch and the validation loss starts at 0_*st*_ epoch with a value of 0.62. It decreases up to 0.0 at 70_*th*_ epoch. Figure 12C shows the confusion matrix of the Bi-LSTM. 63 instances were projected for label 0, 55 instances for C1, 65 instances for C2, and 66 instances for C3 are accurately anticipated. Indicating how successfully the model discriminates across classes, the curve displays the trade-off between sensitivity and specificity. It is better if the performance curve is closer to the upper-left corner. Here, categorization is done using four classifications. The ROC curve for each class of the Bi-LSTM model is displayed in Figure 12D. The studies are conducted in four classes. C0 scored 0.98%, C1 scored 0.95%, C2 scored 0.95%, and C3 scored 0.99%.

**Figure 13:**
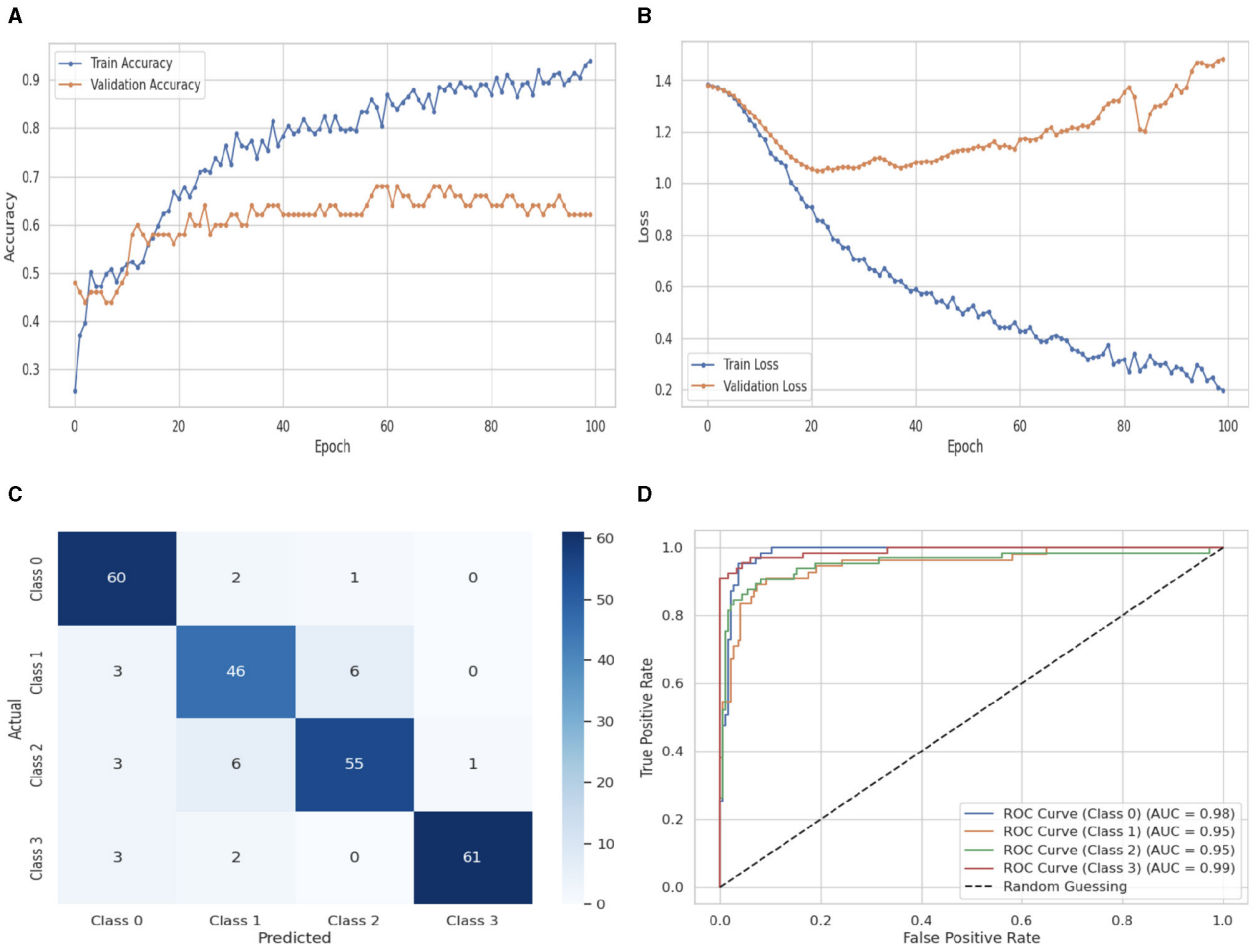
Performance visualization of attention LSTM. **(A)** Train accuracy. **(B)** Train loss. **(C)** CM. **(D)** ROC.

The Attention LSTM classifier's performance evaluation is shown in Figure 13, which also includes CM and ROC graphs, training and validation loss and accuracy. The training and validation accuracy graph is shown in Figure 13A. At 0_*th*_ epoch, the Attention LSTM classifier's training accuracy begins at 0.2%. After some fluctuations increase and decrease, it increases up to 0.92% at 100_*th*_ epoch, and the validation accuracy starts at 0_*th*_ epoch with a value of 0.49%. After some fluctuations increase and decrease and remain constant, it increases up to 0.62% at 100_*th*_ epoch. The training and validation loss graph is shown in Figure 13B. The training loss of the Attention LSTM classifier starts at 0_*th*_ epoch with a value of 1.4. After some fluctuations increase and decrease, it decreases up to 0.2 at 100_*th*_ epoch, and the validation loss starts at 0_*th*_ epoch with a value of 1.4. After some fluctuations, it increases up to 1.6 at 100_*th*_ epoch. Figure 13C displays the Attention LSTM's confusion matrix. The matrix's diagonal cells display the number of cases that were correctly identified, while the off-diagonal cells display the percentage of instances that were incorrectly classified. The predicted labels are represented by the class labels 0, 1, 2, and 3. Sixty instances were projected for label 0, 46 instances for C1, 55 instances for C2, and 61 instances for C3 are accurately anticipated. Indicating how successfully the model discriminates across classes, the curve displays the trade-off between sensitivity and specificity. It is better if the performance curve is closer to the upper-left corner. Here, categorization is done using four classifications. The ROC curve for each class of the Attention LSTM model is displayed in Figure 13D. The studies are conducted in four classes. C0 scored 1.00%, C1 scored 1.00%, C2 scored 1.00%, and C3 scored 1.00%. The area under the curve for all classes is 100% in the Attention LSTM classifier.

### 4.1 Discussion and findings

The performance variations between different classifiers and classes can be attributed to several factors. The Attention LSTM model's superior performance in binary classification may result from its ability to capture temporal dependencies and contextual information in the data effectively. This model focuses on relevant parts of the data, allowing it to handle the intricate nature of the VR dataset better than simpler models. In contrast, simpler models like SVC, KNN, and LR may struggle with the complex, high-dimensional nature of the VR dataset, leading to lower performance. The 100% accuracy achieved by the DNN and Attention LSTM models in multi-class classification suggests that these models can effectively learn and generalize from the dataset's features. The DNN, with its deep architecture, excels in capturing complex data relationships, achieving high accuracy and F1 scores in multi-class classification. However, the high performance might also indicate overfitting, necessitating further validation with diverse datasets to ensure robustness.

The performance disparities observed among different classifiers and across various classes in this study can be attributed to several factors. The Attention LSTM model's superior performance in binary classification stems from its adeptness in capturing temporal dependencies and contextual nuances within the VR dataset. By focusing on salient data components, the Attention LSTM effectively manages the dataset's intricate nature, which simpler models like SVC, KNN, and LR struggle with due to their limitations in handling high-dimensional and complex data. Conversely, deep learning models such as DNN and Attention LSTM achieve notable success in multi-class classification, evident from their 100% accuracy, showcasing their capability to generalize and learn intricate dataset features. However, these models may exhibit signs of overfitting, necessitating validation across diverse datasets to ensure robust performance.

For instance, the SVC classifier, effective in smaller datasets, faltered when faced with high-dimensional VR data, resulting in reduced accuracy and precision. KNN's performance was adversely affected by noise sensitivity and dataset size, impacting its recall and F1 scores. LR, while straightforward, lacked the sophistication to capture nuanced patterns, leading to moderate performance across metrics. Bi-LSTM is proficient in sequential data handling and has improved recall but at the cost of increased computational intensity. These algorithmic differences, coupled with architectural complexities and dataset characteristics, collectively contribute to the performance variations observed across classifiers and classes. The superior performance of DNN and Attention LSTM underscores their ability to discern and exploit intricate data patterns effectively.

Ethical considerations are paramount when utilizing physiological and behavioral data for emotion recognition in virtual reality (VR) environments. This study acknowledges the sensitive nature of such data, including electrocardiogram (ECG), galvanic skin response (GSR), and eye-tracking information, which can provide deep insights into an individual's emotional state. It is essential to ensure that the collection, storage, and utilization of this data adhere to rigorous ethical standards to protect participants' privacy and rights.

Firstly, informed consent procedures were meticulously followed during data acquisition from the VREED dataset, which involved 34 healthy individuals participating voluntarily through focus groups and pilot experiments. Participants were informed about the nature of data collection, the purpose of the study, and their rights regarding data privacy and confidentiality. This transparency is crucial in establishing trust and ensuring that participants understand how their data will be used. Secondly, measures were implemented to safeguard user privacy throughout the data handling process. Data anonymization techniques were employed to remove personally identifiable information, ensuring that individual identities remain protected. Additionally, strict protocols were followed to secure data storage and transmission, utilizing encryption methods and access controls to prevent unauthorized access.

Furthermore, ethical guidelines were adhered to in the processing and analysis of physiological and behavioral data. Data handling procedures were designed to minimize risks of data breaches or misuse. The study also considered potential biases in the dataset and addressed them through careful selection and preprocessing of features, aiming to enhance fairness and equity in emotion recognition outcomes across diverse demographic groups. In conclusion, while leveraging physiological and behavioral data offers promising insights for emotion recognition in VR, it is imperative to uphold ethical principles rigorously. This includes obtaining informed consent, ensuring data anonymity and security, and mitigating potential biases. By adhering to these ethical considerations, this study strives to advance emotion recognition research responsibly and ethically.

### 4.2 Limitations and future direction of the study

One limitation of the current study is the initial simplification of the dataset into binary and multi-class categories, which might have resulted in the loss of nuanced emotional information, potentially affecting the model's ability to recognize subtle variations in emotions. Additionally, while processing for missing values and normalization was necessary, it might have introduced biases or inaccuracies in the dataset, impacting the overall performance of the models. The complexity of the Attention LSTM model, despite its high performance, poses challenges for interpretability and real-world deployment, particularly in understanding the decision-making process of the model. Furthermore, the study's focus on a specific dataset within a controlled VR environment might limit the generalizability of the findings to other settings or real-world scenarios where data variability is higher. Lastly, the deployment of deep learning models, especially those involving attention mechanisms, requires significant computational resources, which might be a constraint in resource-limited environments.

Future research could enhance the current study by incorporating additional modalities such as physiological signals (e.g., heart rate, skin conductance) and voice analysis. These modalities could provide a more comprehensive understanding of emotions and improve model accuracy and robustness. Developing real-time emotion recognition systems for VR applications is another important direction, as it could enhance user experiences and provide immediate feedback. This requires optimizing models for speed and efficiency without compromising accuracy. Ensuring that emotion recognition systems are compatible across various VR platforms and devices is crucial for broader applicability. Future work should focus on creating adaptable models that maintain performance across different hardware and software environments.

To address the complexity and interpretability issues of current models, future research could explore more interpretable machine learning techniques or develop methods to make deep learning models more transparent. Collecting and utilizing larger, more diverse datasets that encompass a wide range of emotional expressions and contexts can help improve the generalizability of the models. This includes data from different demographics, cultures, and settings to ensure the models are robust and inclusive. Finally, conducting user-centric studies to assess the practical implications and usability of emotion recognition systems in real-world VR applications will provide valuable insights into their effectiveness and areas for improvement.

## 5 Conclusion

This study presented an innovative approach for emotion recognition in VR environments using ML and DL classifiers, leveraging a multimodal dataset including eye-tracking and physiological data. Our methodology successfully outperformed traditional approaches in binary and multi-class classification tasks, with the Attention LSTM model excelling in binary classification and the DNN combined with Attention LSTM achieving up to 99.99% accuracy in multi-class scenarios. Despite these advancements, our approach has limitations worth noting: the initial dataset simplification into binary and multi-class categories may overlook subtle emotional distinctions, and while rigorous preprocessing was conducted, potential biases introduced during data handling could impact model performance. Furthermore, the computational demands of deep learning models, especially those with attention mechanisms, pose challenges for real-time deployment in VR settings. Future research will explore integrating additional modalities like voice analysis and developing real-time systems to enhance applicability across diverse VR environments, aiming to improve both the accuracy and practical usability of emotion recognition systems in VR.

## Data Availability

The original contributions presented in the study are included in the article/supplementary material, further inquiries can be directed to the corresponding author.
